# Factors favoring regain of the lost vertical spinal height through posterior spinal fusion in adolescent idiopathic scoliosis

**DOI:** 10.1038/srep29115

**Published:** 2016-07-04

**Authors:** Benlong Shi, Saihu Mao, Leilei Xu, Xu Sun, Zhen Liu, Zezhang Zhu, Tsz Ping Lam, Jack CY Cheng, Bobby Ng, Yong Qiu

**Affiliations:** 1 Spine Surgery, the Affiliated Drum Tower Hospital of Nanjing University Medical School, Nanjing, China; 2 Joint Scoliosis Research Center of the Chinese University of Hong Kong & Nanjing University, Nanjing, China; 3 Department of Orthopaedics and Traumatology, Chinese University of Hong Kong, Hong Kong, China

## Abstract

Height gain is a common beneficial consequence following correction surgery in adolescent idiopathic scoliosis (AIS), yet little is known concerning factors favoring regain of the lost vertical spinal height (SH) through posterior spinal fusion. A consecutive series of AIS patients from February 2013 to August 2015 were reviewed. Surgical changes in SH (ΔSH), as well as the multiple coronal and sagittal deformity parameters were measured and correlated. Factors associated with ΔSH were identified through Pearson correlation analysis and multivariate regression analysis. A total of 172 single curve and 104 double curve patients were reviewed. The ΔSH averaged 2.5 ± 0.9 cm in single curve group and 2.9 ± 1.0 cm in double curve group. The multivariate regression analysis revealed the following pre-operative variables contributed significantly to ΔSH: pre-op Cobb angle, pre-op TK (single curve group only), pre-op GK (double curve group only) and pre-op LL (double curve group only) (*p* < 0.05). Thus change in height (in cm) = 0.044 × (pre-op Cobb angle) + 0.012 × (pre-op TK) (Single curve, adjusted R^2^ = 0.549) or 0.923 + 0.021 × (pre-op Cobb angle_1_) + 0.028 × (pre-op Cobb angle_2_) + 0.015 × (pre-op GK)-0.012 × (pre-op LL) (Double curve, adjusted R^2^ = 0.563). Severer pre-operative coronal Cobb angle and greater sagittal curves were beneficial factors favoring more contribution to the surgical lengthening effect in vertical spinal height in AIS.

A curved spine resulting from a combination of disc and vertebral wedging, rotation and translation, is significantly associated with lost of vertical spinal height^1^,^2^. This secondary short stature could be a cosmetic challenge especially in patients with adolescent idiopathic scoliosis (AIS)^3^. Following correction surgery, some regain of the lost spinal height can be easily noticed by both patients and their parents. This lengthening effect is regarded largely as a simple mechanical phenomenon. However, spinal surgeons are often uncertain about how much height will be gained back after the corrective surgery when being asked.

Several attempts have been made to identify the underlying factors that could affect the surgical spinal height gain. Significant correlation between correction of the Cobb angle and the increase in spinal longitudinal length was recognized in previous studies^4^,^5^,^6^,^7^. However, controversies still exist concerning the influence of sagittal profile^5^,^6^, as well as other features such as the length of the curve, number of instrumented vertebrae and curve pattern. Hwang’s study^5^ attempted to establish the correlation between change in sagittal curvature with the surgical spinal lengthening. The study was, however, limited by the delayed post-operative height measurements which might affect the result. The goals of the current study were: (1) to evaluate the increase in spinal longitudinal length; and (2) to identify the key pre-operative factors that could affect the regain of the lost vertical spinal height following posterior spinal instrumentation and fusion in AIS.

## Materials and Methods

### Subjects

This study was approved by the ethics review board of the affiliated Drum Tower Hospital of Nanjing University, and the methods were carried out in accordance with the guidelines and details from the ethics review board. AIS patients treated surgically from February 2013 to August 2015 in our center were retrospectively reviewed. The inclusion criteria were: (1) AIS patients undergoing only posterior spinal correction and fusion (Lenke 1–3 curves); and (2) with both the pre-operative and immediately post-operative full-length standing antero-posterior (AP) and lateral radiographs (first post-operative erect radiographs obtained before hospital discharge). The exclusion criteria included: (1) patients undergoing posterior osteotomies for the correction of scoliosis; (2) the use of halo traction before or in staged correction surgery. In addition, the patients were stratified into different groups according to the number of complete curves, defined as those curves having symmetrically tilted upper and lower end vertebrae^8^. Only patients with complete curves were included in order to provide better evaluation of the influence of curve pattern on surgical height gain. Informed consents were obtained from all subjects or their parents.

### Radiographic measurements

In our center, the roentgenograms were routinely taken at 1.8 meters distance with a magnification factor of less than 5%. The minimum x-ray dosage for acceptable quality of radiograph for measurement of Cobb angle was used with lead shielding for the breast and gonads (80 ± 10 KV, 40 ± 15 mAs for coronal plain and 100 ± 10 KV, 63 ± 15 mAs for sagittal plain). All the radiographic measurements were performed by the same author (ZL) with more than 5-year experience in spinal surgeon and radiographic assessment on the Picture Archiving and Communications Systems (PACS) workstation (Carestream solution working station, Carestream Health, Version 11.0, Rochester, New York, USA). Pre- and post-operative Cobb angles of major and minor curves were measured from the standing AP x-rays. Correction rate was defined as follows: Correction rate = (pre-op Cobb angle – post-op Cobb angle)/(pre-op Cobb angle) × 100%. In the double curve group, only the Cobb angle of major curve was used in the calculation. The pre- and post-operative spinal height (SH) of each subject was obtained by measuring the vertical distance from the horizontal line crossing the midpoint of T1 upper endplate to the horizontal line crossing the midpoint of L5 inferior endplate. ΔSH was defined as the difference between pre- and post-operative SH (post-op value minus pre-op value). The number of fused levels was also recorded.

On standard standing lateral x-rays (knees and hips in full extension, arms forward directed at 90° and resting on a support), the thoracic kyphosis (TK, the angle between the superior endplate of T5 and the inferior endplate of T12), lumbar lordosis (LL, the angle between the superior endplate of T12 and the endplate of S1) and global kyphosis (GK, the angle between the maximally tilted upper and lower end vertebrae in the sagittal plane, using the standard Cobb’s method) were measured, respectively^9^. Kyphosis was recorded as positive for TK and GK, while lordosis was regarded as positive for LL.

### Statistical analyses

Data were statistically analyzed with the SPSS software 17.0 (SPSS, Inc, USA). Descriptive statistics was performed to analyze the patients’ demographics and data were shown as the mean ± standard deviation (SD). The paired t test was used to compare the mean difference between pre- and post-operative measurements. Univariate Pearson correlation analysis was performed to analyze the association between ΔSH and the candidate correlated factors. Multivariate regression analysis (stepwise) was utilized to identify which factors contributed significantly to ΔSH. Statistical significance was set at *p* value < 0.05.

## Results

In this retrospective study, 172 AIS patients with single complete major thoracic curves and 104 with double complete curves were included, with a female to male ratio of 5.73:1. The average age was 15.6 ± 3.1 years and the mean Cobb angle of the major curves was 53.6 ± 15.4° (range 40°–100°).

### Comparison between pre- and post-operation

The pre-operative SH averaged 41.6 ± 3.0 cm and improved to 44.0 ± 2.8 cm post-operatively in single curve group (ΔSH = 2.5 ± 0.9 cm) and in the double curve group, from average of 40.0 ± 3.3 cm to 42.9 ± 3.0 cm (ΔSH = 2.9 ± 1.0 cm). Paired sample t test revealed significant pre- to post-operative changes in SH (*p* < 0.001), Cobb angle (*p* < 0.001) and LL (double curve group only, *p* = 0.009). The detailed pre- and post-operative parameters of both single and double curve groups were summarized in Table 1.

### Pearson correlation analysis

When combined in the univariate Pearson correlation analysis, we found statistically significant positive correlations between ΔSH and the following measures: pre-op Cobb angle, pre-op TK, pre-op GK and number of fused levels (all *p* < 0.05). Significantly negative correlations were found for pre-op SH, correction rate, change in TK (single curve group only), change in LL (single curve group only) and change in GK (all *p* < 0.05). The detailed results of Pearson correlation analysis were listed in Table 2.

### Multivariate regression analysis

The multivariate regression analyses (stepwise) were performed to compare the relative contributions of each of the parameters on ΔSH. The pre-operative parameters used for the regression analysis included pre-op SH, pre-op Cobb angle, pre-op TK, pre-op LL, pre-op GK and number of fused levels. The following parameters were found to remain significant in the single curve group: pre-op Cobb angle (*p* < 0.001) and pre-op TK (*p* = 0.003) (Table 3). For the double curve group, the pre-op Cobb angle_1_ (major curve, *p* = 0.003), pre-op Cobb angle_2_ (minor curve, *p* = 0.002), pre-op GK (*p* = 0.008) and pre-op LL (*p* = 0.048) were significant (Table 4). Mathematically, the regression equation can be expressed as: Change in spinal height (in cm) = 0.044 × (pre-op Cobb angle) + 0.012 × (pre-op TK) (Single curve, adjusted R^2^ = 0.549) and 0.923 + 0.021 × (pre-op Cobb angle_1_) + 0.028 × (pre-op Cobb angle_2_) + 0.015 × (pre-op GK)−0.012 × (pre-op LL) (Double curve, adjusted R^2^ = 0.563).

## Discussion

Regain of the lost vertical spinal height have long been recognized as a beneficial consequence following scoliosis correction surgery. Theoretically, the amount of spinal lengthening should be equivalent to the loss of height resulting from the scoliotic deformity^4^,^6^. Several studies have attempted to look into factors that could affect the post-operative gain in spinal height, without consistent results. In this study, a multivariate linear model was added into the analysis to allow for more robust analysis.

It is well accepted that differences in surgical strategies and maneuvers could influence the spinal lengthening effect. Watanabe *et al*.^4^ demonstrated that the average increase in vertical spinal height following anterior correction surgeries was approximately half of that in posterior correction surgeries. Besides, the height gain had weak univariate correlation with the correction of Cobb angle^4^. The shortening effect of thorough discectomy, as well as application of maximum compression force during the anterior procedures, was believed to account for such difference. Moreover, adjuvant use of osteotomies could significantly enhance both the correction of scoliosis and the spinal lengthening effect. According to Hwang’s^5^ data, the lengthening effect could exceed the shortening effect of osteotomy resulting from removal of cortical and cancellous bone at the osteotomy vertebra. Thus, in an attempt to limit confounding factors from different surgical strategies, only patients undergoing posterior correction surgeries without osteotomy within tight time interval were included in the current study.

Our study attempted to evaluate the surgical spinal height gain in AIS and to provide improved information of factors that could influence the regaining of the lost vertical spinal height. Parameters associated with the change in spinal height included pre-operative coronal and sagittal profiles of the spine, the corresponding correction obtained post-operatively and number of fused levels. Since the correction in coronal curve was represented by both the severity of scoliosis and the degree of surgical correction, the pre-operative coronal Cobb angles could largely determine the ceiling height gain.

As for the sagittal profile, Sarlak and Watanabe’s studies were limited by the fact that the average changes in kyphosis were relatively small^4^,^7^. Spencer’s study^6^ confirmed that changes in kyphosis and lordosis were small and were non-significant predictors of height gain. However, Hwang *et al*.^5^ have subsequently shown that change in total sagittal curvature could contribute significantly to change in body height. In this study, the spinal height gain was positively correlated with pre-operative thoracic kyphosis and pre-operative global kyphosis and negatively correlated with the change in thoracic kyphosis and global kyphosis in the Pearson correlation analysis. The effect of change in lumbar lordosis was only noticed to be significant in the single curve group. When combined in the multivariate regression analysis, the pre-operative sagittal parameters (pre-op thoracic kyphosis for single curve group, pre-op global kyphosis and lumbar lordosis for double curve group) remained statistically significant. This spinal lengthening effect of the sagittal profile was, according to previous studies, more prominent in patients with kyphotic deformity, such as those with thoracolumbar kyphosis secondary to advanced ankylosing spondylitis^10^,^11^. It is believed that corrections in the sagittal plane could contribute to surgical spinal height changes, though to a lesser degree when compared to that of the coronal plane. We speculated that the recruitment of major lumbar curves (Lenke 5–6 curves) in Spencer’s study might be responsible for the apparent poor correlation between spinal height gain and the sagittal profile. The significantly different contributions of lumbar lordosis on post-operative spinal height gain in single and double curve group were attributed to the differences in fused levels. Notably, over correction of global kyphosis, namely loss of normal sagittal alignments, could lead to increased complications in longer term follow-up in AIS patients.

As for other influencing factors, the number of fused levels was confirmed to be independently related to spinal height gain associated with surgery in previous studies^5^,^6^. Spencer *et al*.^6^ believed that the number of fused levels was the strongest predictor. In this study, the number of fused vertebrae was not found to be significant in the multivariate regression analysis. in clinical practice, the increased surgical height gain from longer fusion should be carefully balanced against the loss in spinal mobility and the long term effect of fusion on adjacent remaining mobile segments.

The negative effect of pre-operative stature on surgical height gain was reported in Spencer’s study^6^. It is worth noting that in Spencer’s study^6^ the degree of curve correction was also included in the predictive model, which would preclude a proper pre-operative prediction in clinical practice. In the current study, only pre-operative coronal and sagittal parameters were retained in the predictive models. In addition to the relatively high adjusted R^2^ values, our predictive formulae could provide better prediction of post-operative spinal height gain.

In addition to the short term effect of post-operative height gain, the long term effect was reported to be restriction of growth of the instrumented spine, which was one of the major concerns in surgical decision-making, especially for adolescents with great growth potential. Hsu *et al*.^12^ studied the effect of spinal fusion on growth of the spine and lower limbs in AIS girls. They revealed that girls treated by posterior spinal fusion showed similar standing height, similar arm lengths, shorter spinal lengths and greater leg lengths at maturity compared with AIS girls treated with a brace, indicating that spinal fusion did retard the longitudinal growth of the spine. However, the loss in spinal length was compensated by an increase in leg length. Interestingly, similar anthropometric characteristics were observed in patients with spinal deformities secondary to tuberculosis^13^. Thus compensatory stimulatory growth mechanisms leading to longer legs and upper limbs in patients with stunted spinal growth in childhood before skeletal maturity was possible. Spencer *et al*.^6^ further showed a small but significant increase of 4.6 mm in spinal height occurred between the immediate post-operation and two years follow-up. The younger age at time of surgery, male sex, fewer levels fused, and a preoperative Risser stage of ≤2 were independent predictors of spinal growth at two years follow-up.

Limitations of this study included, firstly, no patients with three complete curves were recruited. Secondly, we believed the predictive equations could serve as reasonable estimation but not at precision. Thirdly, the spinal height was measured from T1 to L5 in the current study, which was a little different from the SRS protocol (T1-S1). Besides, no inter- and intro-observer reliability study was conducted on the measurement of spinal height. In addition, it should be mentioned that emphasis on radiographic spinal height gain should not deviate from the original aim of the surgery, namely to prevent progression and to rebalance the spine three-dimensionally. Lastly, plain radiographs were known to have limitations for the assessment of upper thoracic vertebra due to the overlap of the shoulder girdle and EOS imaging system^14^,^15^ would be highly recommended in future studies when available. Therefore, further related studies are suggested to include larger sample size, more curve patterns and EOS data, and be with reference to the standard SRS protocol.

In conclusion, this study provided an improved understanding for factors favoring regain of the lost vertical spinal height. Severer pre-operative coronal Cobb angle and greater sagittal curves were beneficial factors favoring more contribution to the surgical lengthening effect in spinal height in AIS.

## Additional Information

**How to cite this article**: Shi, B. *et al*. Factors favoring regain of the lost vertical spinal height through posterior spinal fusion in adolescent idiopathic scoliosis. *Sci. Rep*. **6**, 29115; doi: 10.1038/srep29115 (2016).

## Figures and Tables

**Table 1:** Summary of pre- and post-operative parameters.

	Single Curve group			Double Curve group		
	Mean ± SD	Range	*p*	Mean ± SD	Range	*p*
Pre-op SH(cm)	41.6 ± 3.0	33.1–49.8	<0.001	40.0 ± 3.3	30.9–47.3	<0.001
Post-op SH(cm)	44.0 ± 2.8	37.0–51.7		42.9 ± 3.0	34.9–50.6	
Pre-op Cobb angle_1_(°)	51.9 ± 14.2	40–140	<0.001	56.6 ± 16.8	40–135	<0.001
Post-op Cobb angle_1_(°)	15.7 ± 9.2	3–79		19.5 ± 13.1	4–95	
Pre-op Cobb angle_2_(°)	—	—	—	37.3 ± 13.1	17–84	<0.001
Post-op Cobb angle_2_(°)	—	—		15.0 ± 9.4	2–56	
Pre-op TK(°)	20.3 ± 13.6	−14–78	0.370	19.0 ± 11.1	0–61	0.852
Post-op TK(°)	21.0 ± 9.0	1–55		19.3 ± 7.5	3–37	
Pre-op LL(°)	50.8 ± 12.4	−3–84	0.529	52.9 ± 12.1	17–90	0.009
Post-op LL(°)	50.0 ± 10.2	27–80		50.0 ± 11.5	7–76	
Pre-op GK(°)	27.2 ± 14.3	−16–102	0.201	26.2 ± 13.9	4–87	0.801
Post-op GK(°)	28.3 ± 8.9	10–55		25.9 ± 8.2	3–43	
Correction rate of major curve (%)	70.7 ± 10.2	43.6–92.9	—	67.2 ± 11.9	29.6–91.9	—
Change in SH(cm)	2.5 ± 0.9	0.5–7.2	—	2.9 ± 1.0	1.2–6.7	—
Change in TK(°)	0.7 ± 10.6	−40–26	—	0.4 ± 11.4	−28–29	—
Change in LL(°)	−0.6 ± 11.7	−41–47	—	−2.5 ± 14.0	−30–68	—
Change in GK(°)	1.1 ± 11.2	−58–38	—	0.1 ± 14.4	−24–53	—
Number of fused levels	10.9 ± 2.4	5–16	—	12.7 ± 1.3	7–16	—

Abbreviation: Spinal height (SH), thoracic kyphosis (TK), lumbar lordosis (LL), global kyphosis (GK).

**Table 2:** Univariate Pearson correlation analysis with ΔSH in single and double curve groups.

	Single Curve group		Double Curve group	
	Coefficient	*p*	Coefficient	*p*
Pre-op SH	−0.434	<0.001	−0.363	<0.001
Pre-op Cobb angle_1_	0.722	<0.001	0.707	<0.001
Pre-op Cobb angle_2_	—	—	0.629	<0.001
Pre-op TK	0.453	<0.001	0.238	0.017
Pre-op LL	0.124	0.109	−0.150	0.134
Pre-op GK	0.366	<0.001	0.304	0.002
Correction rate of major curve	−0.252	0.001	−0.417	<0.001
Change in TK	−0.389	<0.001	−0.195	0.050
Change in LL	−0.198	0.010	−0.059	0.555
Change in GK	−0.318	<0.001	−0.391	<0.001
Number of fused levels	0.329	<0.001	0.550	<0.001

Abbreviation: Refer to Table 1.

**Table 3:** Multivariate regression analysis with ΔSH in single curve group.

Model	Parameters	B-Coefficient	*p*
1	Constant	−0.051	0.788
	Pre-op Cobb angle	0.048	<0.001
2	Constant	−0.041	0.826
	Pre-op Cobb angle	0.044	<0.001
	Pre-op TK	0.012	0.003

Abbreviation: Refer to Table 1.

**Table 4:** Multivariate regression analysis with ΔSH in double curve group.

Model	Parameters	B-Coefficient	*p*
1	Constant	0.486	0.055
	Pre-op Cobb angle_1_	0.042	<0.001
2	Constant	0.434	0.074
	Pre-op Cobb angle_1_	0.025	0.001
	Pre-op Cobb angle_2_	0.028	0.002
3	Constant	0.290	0.244
	Pre-op Cobb angle_1_	0.021	0.004
	Pre-op Cobb angle_2_	0.030	0.001
	Pre-op GK	0.010	0.045
4	Constant	0.923	0.023
	Pre-op Cobb angle_1_	0.021	0.003
	Pre-op Cobb angle_2_	0.028	0.002
	Pre-op GK	0.015	0.008
	Pre-op LL	−0.012	0.048

Abbreviation: Refer to Table 1.
